# Factors Associated with Intention to Donate Blood: Sociodemographic and Past Experience Variables

**DOI:** 10.1155/2014/571678

**Published:** 2014-11-05

**Authors:** Pule Ishmael Pule, Boitshwarelo Rachaba, Mgaywa Gilbert Mjungu Damas Magafu, Dereje Habte

**Affiliations:** Faculty of Medicine, University of Botswana, Private Bag 00713, Gaborone, Botswana

## Abstract

*Background and Objectives*. This study was conducted to assess the level of intention of the general public towards blood donation and the factors associated with it. *Methods*. A descriptive cross-sectional study was conducted in South-East Botswana amongst participants aged 21–65 years. An interviewer-administered questionnaire was completed for 384 participants. *Results*. Of the 384 participants, 104 (27.1%) reported that they had donated blood in the past and 269 (70.1%) stated that they were willing to donate blood in the future. Thirteen out of the 104 past donors (12.5%) reported that they had donated blood in the 12 months preceding the survey and only 10 (9.6%) participants reported that they have been regular donors. In the backward logistic regression analysis, the variables that remained significant predictors of the intention to donate blood were secondary education (adjusted odds ratio (AOR) (95% confidence interval (CI)): 2.92 (1.48, 5.77)), tertiary education (AOR (95% CI): 3.83 (1.52, 9.62)), and knowing a family member who had ever donated blood (AOR (95% CI): 2.84 (1.58, 5.12)). *Conclusion*. Being informed about blood transfusion and its life-saving benefits through either the education system or the experience made people more likely to intend to donate blood. Evidence-based interventions to retain blood donors as regular donors are recommended.

## 1. Introduction

Blood transfusion is becoming a crucial component in the management of patients presenting with accident injuries, surgical conditions, malignancies, pregnancy complications, and other medical conditions [1, 2]. In high income countries, the major indications for transfusion include sophisticated medical and surgical procedures, malignancies, and trauma. Pregnancy complications and childhood anemia are the conditions that largely need blood transfusion in middle and low income countries. More than one-quarter of maternal deaths could be averted by having access to safe blood [2–4].

WHO estimates that at least 1% of the population needs to donate blood to meet the minimum requirement of blood for a country [1]. Globally, 70 countries have a blood donation level less than the optimal level of 10/1000 population [1]. The African continent managed to collect blood to satisfy only 41% of the demand in 2006 [5, 6]. The gap between supply and demand for blood is wider in developing and transitional countries than in developed counterparts [1, 2]. According to Botswana National Blood Transfusion Services (NBTS), the country needs 36,000 units of blood annually. Records show that 23,275 units of blood were collected in the year 2009 followed by a reduction to 20,401 units collected in 2010 and 16,562 units collected in 2011 (unpublished report by Botswana NBTS, 2012).

Altruism, social responsibility, peer influence, access to health communication, and knowledge about importance of blood donation are mentioned as some of the factors that motivate individuals to donate blood [7–12]. Transmission of values to generations among family members practicing donation and the influence of active blood donors on others are also noted [12, 13]. The retention of blood donors as regular donors is critical to ensure regular supply of blood which is influenced by a range of factors, namely, demographic, psychosocial, altruism, social obligation, prior donation frequency, satisfaction with the last donation experience, and behavioral factors [14–18].

Studies have demonstrated that the intention to donate blood predicts the practice of blood donation [18, 19]. Demographic, knowledge status, and behavioral factors are shown to determine individuals' intention to donate blood [20–22]. Hence, it is worthwhile to study the intention of community members for blood donation in Botswana to understand the situation and come up with evidence-based interventions. This study was conducted to assess the level of intention of the general public in South-East Botswana towards blood donation and the factors associated with the intention.

## 2. Methods

### 2.1. Study Site and Study Population

A descriptive cross-sectional study was conducted in Kweneng district in South-East Botswana. The population of the district was estimated to be 304,674 with a density of 6.4 people per square kilometer [23]. The study participants were recruited from Molepolole village which is the capital of Kweneng district with a population of 67,598 [23]. The study was conducted amongst members in the selected households in Molepolole. They were aged 21–65 years irrespective of gender.

### 2.2. Sample Size and Sampling

Epi-Info software version 3.5.3 (US CDC, Atlanta, Georgia) was utilized to compute the sample size. The proportion of the intention to donate blood was assumed to be 50% to attain the maximum sample size. With a margin of error of 5% at 5% level of significance, a sample size of 384 was determined. EPI-random walk method [24] was used to select the households. The starting point was selected at Kgosing, the office for local leaders (at the center of the village). A bottle was spun and households along the direction of the bottle top were included in the data collection. One eligible member in each selected household was interviewed until reaching the required sample size. If there were more than one eligible person in the selected household, only one randomly selected participant was included using lottery method.

### 2.3. Data Collection and Analysis

A questionnaire was developed in English after a thorough literature review to include the relevant variables. It was then pilot tested and validated. Two enumerators trained on the questionnaire collected the data. The principal investigators were involved in the supervision of the data collection. Data were collected between August 27 and September 21, 2012. In this study, a regular blood donor was a person who voluntarily donated blood routinely, that is, 2–4 times a year [1].

Data were entered using Epi-Info software version 3.5.3 and exported to SPSS version 20 (IBM, NY, USA) for analysis. Frequency, percentage, and mean were computed to describe the findings. The crude and adjusted odds ratio (COR/AOR) and 95% confidence intervals (CI) were analyzed to explore associations. Backward logistic regression analysis was employed to control the effect of confounding variables. *P* values less than 0.05 were considered statistically significant.

### 2.4. Ethical Issues

Ethical approval was secured from the Institutional Review Boards of the University of Botswana and the Ministry of Health Research Unit of Botswana. The District Health Management Team and the local administrators granted permission to conduct the research. Signed consent was obtained from all participants before the conduct of the interview. No personal identifying details were recorded on the questionnaire.

## 3. Results

A total of 384 participants were included in the study with female to male ratio of 1.46. Three-fourths of the participants were in the age range of 21–40 years and 73.9% had educational level of secondary school and above. Married and singles accounted for 17.4% and 81% of the sample, respectively. The vast majority (80.2%) were followers of Christianity while 15.1% were not followers of any religion (Table 1). The younger the age of the study participants, the better their educational achievement (*P* = 0.00).

**Figure 1:**
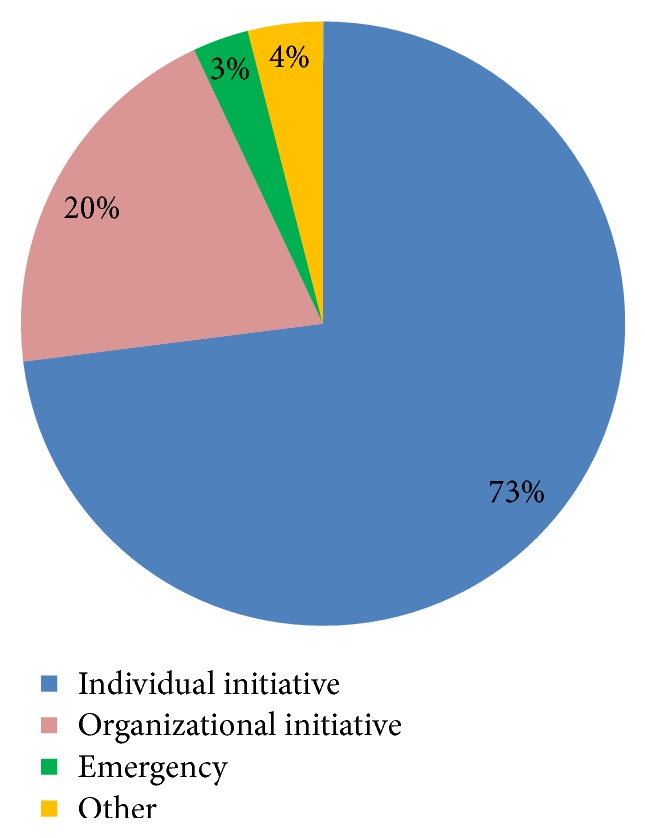
Reasons for blood donation among donors (*n* = 104).

**Figure 2:**
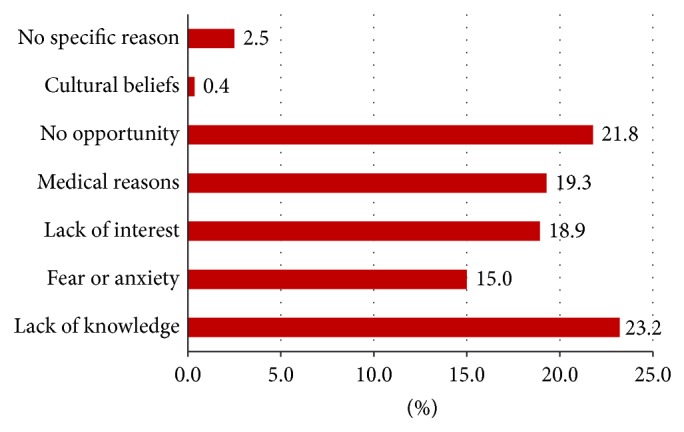
Reasons for not donating blood among nondonors.

Over four-fifths of the study participants (327) had heard about blood donation in the past of which 104 (31.8%) have ever donated blood. The major sources of information on blood donation were school (35.8%), health facilities (26.6%), and the media (30.6%). Most participants knew at least one condition that may need blood transfusion as a treatment. Of the 384 participants, 104 (27.1%) reported that they had donated blood in the past and 269 (70.1%) stated that they were willing to donate blood in the future. Thirteen out of the 104 past donors (12.5%) reported that they had donated blood in the 12 months preceding the survey and only 10 participants (9.6%) reported that they had been regular donors (Table 2). The reasons for blood donation among donors were largely individual initiative (73%) and organizational initiative (20%) (Figure 1). Among the participants who never donated blood, lack of knowledge, absence of opportunities, medical reasons, lack of interest, and fear were among the reasons listed for not donating blood (Figure 2).

Only 31% reported that they came across a close family member who had ever donated blood. Similarly, 26.8% reported that they knew a friend who had donated blood in the past. Participants whose close family member had ever needed blood transfusion in the past constituted 116 (30.2%), of which 97 (83.6%) managed to receive the transfusion. Unavailability of blood, cultural reasons, and patient death were amongst the reasons for the patients' failure to receive blood transfusion (Table 3).

Table 4 shows the COR and AOR for the different exposure variables versus willingness to donate blood. A backward logistic regression analysis was made to compute AORs and only two variables were retained in the final regression model. In the bivariate analysis, younger age group and secondary and tertiary educational levels were amongst the sociodemographic variables that showed significantly increased willingness as compared to the reference groups. Other exposure measures that showed significantly increased association in the bivariate analysis included prior history of blood donation (COR (95% CI): 1.99 (1.17, 3.43)), knowing a family member who ever donated blood (COR (95% CI): 3.52 (1.99, 6.23)), and knowing a friend who ever donated blood (COR (95% CI): 3.24 (1.78, 5.90)). In the backward logistic regression analysis, the variables that remained significant predictors of willingness to donate blood were secondary education (AOR (95% CI): 2.92 (1.48, 5.77)), tertiary education (AOR (95% CI): 3.83 (1.52, 9.62)), and knowing a family member who had ever donated blood (AOR (95% CI): 2.84 (1.58, 5.12)).

## 4. Discussion

This study was a community-based study representing one of the urban villages in Botswana. Findings from a community-based study are more representative of the general public's intention to donate blood than findings from a facility-based study. The study population was homogeneous in terms of marital status, religion, and residence of participants whereby the large majority were single, Christians, and urban residents. Such homogeneity is thought to act as a self-control mechanism for potential confounding that may be caused by certain variables. The Botswana Demographic Survey also demonstrated a similar distribution in relation to gender, marital status, and religion [25]. Hence, the study should be considered to have acceptable representation of the population studied.

The main finding of this study is that better educational background and exposure to past donors were the predictors of intention to donate blood. Both younger age group and better educational status were factors that were significantly associated with the intention to donate blood in the bivariate analysis. However, only the latter turned out to be statistically significant in the multivariable analysis. The result demonstrated that the educational status of younger participants was better than that of their older counterparts. Hence the higher willingness to donate blood among the younger age groups may be attributed to the direct effect of the better educational background of the younger age groups rather than to their age. Individuals who reported that they had donated blood in the past and who reported that they knew someone who had ever donated blood were more likely to intend to donate blood in the future, results which are in conformity with studies done in different settings [12, 13, 20, 21]. In contrast, another study reported that blood drive organizers and/or recruiters were more important than family and/or peers in encouraging donors [11]. It is likely that witnessing blood donors without any complication following the procedure improves the confidence and disproves misconceptions among community members. The influence of family and other active blood donors on their contacts is also demonstrated in previous studies [12, 13]. Programs need to consider close family members and friends who donated blood in the past as change agents in community blood donation mobilization efforts.

A total of 327 study participants reported that they have heard about blood donation of which 104 (31.8%) have ever donated blood. A significant proportion of participants had heard about blood donation which was not reflected in relation to the practice of blood donation in the past. It shows that information alone is not sufficient and behavior change communication approach needs to be employed to guarantee blood donation practice. Blood transfusion centers, the Red Cross Society, and blood donation campaigns were cited as sources of information by few participants. Moreover, public service centers like offices, kgotlas (local leaders' institutions), and churches were cited as information sources by the minority. It is quite important to diversify health education efforts by including such centers in order to bring about the desired behavior change especially through the involvement of community and religious leaders. Religious leaders were demonstrated to have an influential role in studies done among African Americans and the Middle East [26–28].

Over a quarter of the participants reported that they have ever donated blood which is a good proportion if they were retained as regular blood donors. The level of those who had ever donated blood from one study in India ranged from 7.7% to 12.7% [29, 30] whereas two-thirds of the participants reported that they have ever donated blood in studies done in the Middle East and USA [28, 31]. In the current study, only 12.5% of the participants who mentioned that they have ever donated blood reported that they have donated the blood in the 12 months preceding the survey. There is a significant gap in that those who reported to have ever donated blood were not retained to be regular donors. Studies have demonstrated that higher prior donation frequency was a predictor for donor return [18, 19]. Once individuals come for blood donation to the centers, a mechanism to retain them as regular blood donors needs to be devised. WHO and the International Federation of Red Cross and Red Crescent Societies recommend establishing a database of loyal and regular donors as a means of having access to a safe blood supply. This includes having a mechanism for routinely recalling donors so that they can donate every 3-4 months [1].

Intention to donate in the future was reported by more than two-thirds of the participants. Willingness is the starting point for behavior change as was demonstrated by a study done among new and experienced blood donors [18]. It is an indication that the opportunity still exists in terms of intention despite the low level of past practice of donation. However, it is worth noting that not all intending people can be eligible for blood donation in a setting like Botswana with a national HIV prevalence of 16.9%. The HIV prevalence in the age range 30–49 years was reported to be higher than the national average estimate (range: 33.9%–43.7%) [32]. Hence, there is a need to have a larger pool of volunteers to compensate for the potential ineligibility despite individuals' willingness to donate blood. The reasons given for not donating blood in the past are diverse and future interventions need to take into account such factors. In-depth investigation on those factors is of paramount importance to clearly understand the root causes and come up with evidence-based interventions.

It is worth noting the following limitations of this study. We used nonprobability sampling and the presence of sampling bias cannot be ruled out. The sample might not be fully representative of the rural population as the study was conducted in a semiurban setting. The intention to donate blood reported during data collection may not necessarily be genuine, which is one of the limitations of reported responses. This study did not explore the reasons why one-time donors ended up donating only once. Could it be that they had a bad experience at their first donation? Future research studies should explore the reasons why some donors donate blood only once.

## 5. Conclusion

Being informed about blood transfusion and its life-saving benefits through either the education system or the life experiences had made people more likely to intend to donate blood. The intention for future blood donation far outweighs past practice of blood donation. The high level of willingness to donate blood needs to be considered as an opportunity for future community mobilization initiatives. Health programs need to target behavior change using diverse approaches including the use of current blood donors and local leaders as change agents. Evidence-based interventions to retain blood donors as regular donors are of paramount importance. Further studies to understand the root causes among nondonors as well as the reasons behind failure to retain regular blood donors are recommended.

## Figures and Tables

**Table 1:** Background characteristics of study participants.

Characteristic	Frequency (*n* = 384)	Percentage
Age in years		
21–30	187	48.7
31–40	105	27.3
41–50	47	12.2
51 and above	45	11.7
Gender		
Male	156	40.6
Female	228	59.4
Education level		
No formal education	44	11.5
Primary	56	14.6
Secondary	229	59.6
Tertiary	55	14.3
Marital status		
Married	67	17.4
Single	311	81.0
Widowed	2	0.5
Divorced	2	0.5
No response	2	0.5
Religion		
Christianity	308	80.2
Botswana traditional religion	6	1.6
Atheism	7	1.8
Rastafarian	1	0.3
No religion	58	15.1
No response	4	1

**Table 2:** Participants' knowledge and practice of blood donation.

Characteristic	Frequency	Percentage
Ever heard about blood donation (*n* = 384)		
Yes	327	85.2
No	57	14.8
Source of information (*n* = 327)^a^		
School	117	35.8
Health facilities	87	26.6
Media	100	30.6
Blood transfusion center	6	1.8
Red cross	5	1.5
Blood donation campaign	6	1.8
Work place	11	3.4
Family or friends	14	4.3
Public service centers (offices, kgotla, church, and mall)	9	2.8
Knows a health condition that requires blood transfusion (*n* = 384)		
Yes	295	76.8
No	89	23.2
Knowledge on blood donation by gender (*n* = 384)		
Males and females can donate	350	91.1
Only males can donate	34	8.9
Number of possible donations in one year (*n* = 384)		
One	21	5.5
Two	54	14.1
Three	36	9.4
Four	4	1.0
No idea	269	70.1
Ever donated blood (*n* = 384)		
Yes	104	27.1
No	280	72.9
Donated blood in the past 12 months (*n* = 104)		
Yes	13	12.5
No	91	87.5
Status of blood donation (*n* = 104)		
Regular donor	10	9.6
Nonregular donor	94	90.4
Willingness to donate blood in the future (*n* = 384)		
Yes	269	70.1
No	115	29.9

^a^% exceeded 100% as some participants had more than one response.

**Table 3:** Past experiences of blood donation and transfusion among close contacts.

Characteristic	Frequency	Percentage
Knows a close family member who donated blood (*n* = 384)		
Yes	119	31.0%
No	265	69.0%
Knows a friend who donated blood (*n* = 384)		
Yes	103	26.8%
No	281	73.2%
Close family member ever needed blood transfusion (*n* = 384)		
Yes	116	30.2%
No	268	69.8%
Close family member ever got blood transfusion (*n* = 116)		
Yes	97	83.6%
No	19	16.4%
Reason for no blood transfusion (*n* = 19)		
Unavailability of blood	6	31.6%
Cultural reasons	2	10.5%
Patient died soon	5	26.3%
No response	6	31.6%

**Table 4:** Factors associated with the intention to donate blood in the future.

Factor	Willing to donate blood		Crude odds ratio (95% CI)	Adjusted odds ratio (95% CI)
	Yes	No		
Age in years				
21–30	151	36	4.39 (2.20,8.73)^**^	
31–40	70	35	2.09 (1.03,4.26)^*^	
41–50	26	21	1.29 (0.57, 2.94)	
51 and above	22	23	Reference	
Gender				
Male	112	44	Reference	
Female	157	71	0.87 (0.56, 1.36)	
Education level				
No formal education	22	22	Reference	Reference
Primary	25	31	0.81 (0.37, 1.78)	0.81 (0.36, 1.82)
Secondary	177	52	3.40 (1.75, 6.63)^**^	2.92 (1.48, 5.77)^**^
Tertiary	45	10	4.50 (1.82, 11.12)^**^	3.83 (1.52, 9.62)^**^
Ever heard about blood donation				
Yes	235	92	1.73 (0.97, 3.09)	
No	34	23	Reference	
Knows a health condition that requires blood transfusion				
Yes	210	85	1.26 (0.76, 2.09)	
No	59	30	Reference	
Ever donated blood				
Yes	83	21	1.99 (1.17,3.43)^*^	
No	186	94	Reference	
Knows a close family member who donated blood				
Yes	102	17	3.52 (1.99, 6.23)^**^	2.84 (1.58, 5.12)^**^
No	167	98	Reference	Reference
Knows a friend who donated blood				
Yes	88	15	3.24 (1.78, 5.90)^**^	
No	181	100	Reference	
Close family member ever needed blood transfusion				
Yes	86	30	1.33 (0.82, 2.17)	
No	183	85	Reference	

^*^ *P* value less than 0.05; ^**^ *P* value less than 0.01.
